# MRS and Optical Imaging Studies of Therapeutic Response to Combination Therapy Targeting BRAF/MEK in Murine Melanomas

**DOI:** 10.1016/j.acra.2025.01.035

**Published:** 2025-02-20

**Authors:** Pradeep Kumar Gupta, Lin Z. Li, Dinesh Kumar Singh, Skyler Nova, Fernando Arias-Mendoza, Stepan Orlovskiy, Sanjeev Chawla, David S. Nelson, Michael D. Farwell, Kavindra Nath

**Affiliations:** Department of Radiology, Perelman School of Medicine, University of Pennsylvania, Philadelphia, Pennsylvania (P.K.G., L.Z.L., D.K.S., S.N., F.A.M., S.O., S.C., D.S.N., M.D.F., K.N.); Abramson Cancer Center, University of Pennsylvania, Philadelphia, Pennsylvania (L.Z.L., K.N.); Institute of Translational Medicine and Therapeutics, University of Pennsylvania, Philadelphia, Pennsylvania (L.Z.L., K.N.); Advanced Imaging Research, Inc. Cleveland, Ohio (F.A.M.)

**Keywords:** ^1^H and ^31^P Magnetic Resonance Spectroscopy, Optical Redox Imaging, Melanoma, BRAF, MEK

## Abstract

**Rationale and Objectives:**

Melanoma, an aggressive skin cancer, often harbors BRAFV600E mutations driving tumor progression via the mitogen-activated protein kinase (MAPK) pathway. While targeted therapies like BRAF (dabrafenib) and MEK (trametinib) inhibitors have improved outcomes, resistance linked to metabolic reprogramming remains a challenge.

This study investigates metabolic changes induced by dual BRAF/MEK inhibition in a BRAFV600E-mutant murine melanoma model using magnetic resonance spectroscopy (MRS), optical redox imaging (ORI), and biochemical assays. We aim to identify metabolic biomarkers for predicting therapeutic response or resistance.

**Materials and Methods:**

YUMM1.7 murine melanoma cells and tumored mice were treated with dabrafenib and trametinib. ORI assessed mitochondrial redox status by measuring reduced nicotinamide adenine dinucleotide (NADH), oxidized flavoproteins (Fp), and the redox ratio (Fp/(NADH+Fp)) in vitro. Glucose consumption and lactate production were analyzed using a YSI Biochemical Analyzer. In vivo metabolic changes were monitored via ^1^H and ^31^P MRS, evaluating lactate, alanine, pH, βNTP/Pi, and total NAD(P)(H), which represents combined oxidized nicotinamide adenine dinucleotide (NAD^+^), NADH, and reduced nicotinamide adenine dinucleotide phosphate (NADPH).

**Results:**

Under the combined therapeutic regimen of dabrafenib and trametinib, YUMM1.7 murine melanoma cells exhibited significant inhibition of lactate generation, non-significant reduction of glucose utilization, decreased intracellular levels of NADH and total NAD(P) (H), and more oxidized redox status in vitro, which can be interpreted as inhibition of the Warburg effect and improved OXPHOS efficiency by targeting BRAF/MEK signaling activities. Furthermore, YUMM1.7 mouse tumors demonstrated less tissue acidification and improved bioenergetics (βNTP/Pi), in agreement with the in vitro data.

**Conclusion:**

MRS, ORI, and biochemical assays identified critical metabolic changes, highlighting potential biomarkers and supporting the integration of metabolic inhibitors with MAPK-targeted therapies to improve clinical outcomes.

## INTRODUCTION

Melanoma, a malignancy arising from melanocytes, is the deadliest form of skin cancer due to its high potential for metastasis (1). In the United States, melanoma ranks as the fifth most common cancer in men and the sixth in women (2). Over the past two decades, its incidence has increased more rapidly than that of most other cancers, both in the United States and globally (3). Melanoma is highly treatable when detected early and confined to its primary site. However, once it progresses to metastatic disease, the prognosis becomes poor, with a median overall survival of only six to eight months (4). Moreover, current systemic treatments for metastatic melanoma are associated with variable success rates, and tumors often develop resistance to these therapies (5–7). Therefore, the accurate and early diagnosis of melanomas provides a window of opportunity for reframing treatment strategies, enabling optimal and timely therapeutic interventions in these patients.

Although surgical treatment remains the primary mode of treatment for melanomas (8), recent advances in immunotherapies and targeted molecular therapies offer significant promise for managing metastatic melanomas (8). Melanoma cells, which are often resistant to chemotherapy and radiotherapy due to melanin production, are typically treated using multimodal therapy including surgery, chemotherapy, immunotherapy, and radiotherapy. In recent years, targeted therapies have been introduced focusing on mitogen-activated protein kinase MAPK pathway, a critical regulator of various cellular processes (9).

This report examines the response of the genetically engineered murine melanoma model YUMM1.7, which harbors the BRAF V600E mutation to the targeted cancer therapy, i.e., combined dabrafenib and trametinib. The BRAF V600E mutation, a common oncogenic driver of melanoma, constitutively activates the MAPK signaling pathway, leading to uncontrolled cellular proliferation and survival (10). Dabrafenib selectively inhibits mutant BRAF kinase, while trametinib, targets MEK1/2 downstream of BRAF (11,12). Together, these inhibitors disrupt aberrant MAPK signaling and demonstrate synergistic anti-tumor effects by preventing compensatory pathway reactivation, a common mechanism of therapeutic resistance.

In parallel, the study explores the utility of optical redox imaging (ORI) as a highly sensitive method for monitoring tumor metabolism (13–15). ORI, pioneered by Britton Chance and his coworkers, leverages the intrinsic autofluorescence of two key metabolic cofactors: NADH (reduced nicotinamide adenine dinucleotide), which is a predominant regulator of glycolysis, the Krebs cycle, and OXPHOS, and oxidized flavin adenine dinucleotide (FAD) contained in flavoproteins (Fp), which is involved in the Krebs cycle and OXPHOS. By quantifying the optical redox ratio (ORR), defined as Fp/(NADH + Fp) or FAD/(NADH + FAD), ORI provides a real-time, quantitative measure of cellular redox balance, which is closely linked to the NAD^+^/NADH ratio (16,17). Changes in ORR reflect metabolic shifts induced by oncogenic mutations (18), drug treatments (19), or cellular stress (20), making it a powerful biomarker for assessing metabolic adaptation and therapeutic response in cancers.

The in vivo MRS, also pioneered by Britton Chance, provides valuable biochemical and bioenergetic information in vivo and has been extensively applied to cancer research. Our previous studies (11,12) demonstrated that ^1^H and ^31^P MRS could noninvasively monitor key metabolic parameters, including lactate, alanine, pH, and bioenergetics (βNTP/Pi), to capture the effects of single-agent BRAF or MEK inhibition in human melanoma models. In the present study, we will provide a comprehensive assessment of melanoma metabolism by combining ORI and ^1^H and ^31^P MRS techniques, measuring additional critical bioenergetic markers such as NADH, ORR, and the total NAD(P)(H). Together, these complementary methodologies enable detailed profiling of metabolic phenotypes and reveal the metabolic reprogramming induced by therapeutic intervention in both in vitro and in vivo settings.

The primary objective of our study is to assess the therapeutic efficacy and metabolic impact of combined dabrafenib and trametinib treatment in YUMM1.7 melanoma models. Specifically, this study aims to investigate the metabolic effects of dual MAPK pathway inhibition, focusing on alterations in redox status, glycolytic activity, and mitochondrial function, which are critical factors influencing tumor progression and resistance. Furthermore, by integrating ^1^H/^31^P MRS, ORI, and biochemical assays into the experimental framework, this study aims to establish these techniques as valuable tools for metabolic monitoring in cancer research. The anticipated outcome will offer mechanistic insights into the relationship between oncogenic signaling and metabolism, reprogramming, contributing to the development of more effective therapeutic strategies and novel metabolic biomarkers for melanoma for understanding tumor microenvironment (TME) of melanomas and evaluating treatment response.

## MATERIALS AND METHODS

### In Vitro Experiments

#### Melanoma Cell Models, Culturing, and Survival Measurements

YUMM1.7 is a genetically engineered murine melanoma cell line that was obtained from Dr. Marcus Bosenberg’s laboratory, Yale University, New Haven, CT, USA. This cell line harbors a key mutation e.g. BRAF V600E, which collectively drives tumorigenesis and mimics the genetic landscape of the skin of an adult male mouse. These mutations render YUMM1.7 as an ideal model for studying therapeutic responses and exploring targeted treatment strategies. The YUMM1.7 cell line was grown in α-MEM medium supplemented with 25 mmol/L glucose, 2 mmol/L glutamine, 10 mmol/L HEPES, 100 Units/mL penicillin, 100 μg/mL streptomycin, and 10% FBS. YUMM1.7 melanoma cells were seeded in T-75 tissue culture flasks and incubated at 37 °C with 5% CO_2_ for 24 h to allow adhesion. Afterward, either vehicle (0.1% DMSO) or dabrafenib (25 nmol/L) plus trametinib (7 nmol/L) was added to create control and treated groups. Cell survival was measured by trypan-blue staining and manually counted with a hemocytometer under a bright field microscope (21).

#### Drug Treatment

Dabrafenib and trametinib were purchased from LC Laboratories (Woburn, MA, USA). 3-aminopropylphosphonate (3-APP) was purchased from AmBeed (Arlington Heights, IL, USA). The combined dabrafenib (25 nmol/L) plus trametinib (7 nmol/L) or 0.1% DMSO (control) were used to treat cell cultures. For mice, drugs were used in combination i.e. (30 mg/kg dabrafenib + 10 mg/kg trametinib) and were administered by oral gavage daily until day 5. The drugs were dissolved in buffer (0.5% hydroxypropyl methylcellulose plus 0.2% tween 80 in milli-Q water).

#### Extracellular Glucose and Lactate Measurements

After 48 h of treatment, medium samples were collected from both control and treated groups, and extracellular glucose and lactate concentrations were measured using a YSI 2300 STAT PLUS Biochemistry Analyzer (YSI Incorporated, Yellow Springs, OH, USA). The glucose utilization was obtained by subtracting the residual glucose present in the cell-cultured media after 48 h from the glucose concentration present in the culture media at the start of the experiment (basal glucose concentration). The lactate generation was similarly obtained by subtracting the basal lactate concentration present in the media before the start of the cell culture or treatment from the lactate present at the end of the experiment post 48 h. Both the glucose utilization and lactate generation profiles were normalized with respect to cell density in order to calculate the glucose utilization or lactate production per million cells. The experiment was performed in triplicate.

#### Optical Redox Imaging (ORI)

YUMM1.7 melanoma cells (15,000 for control and 20,000 per for treatment) were cultured in 1 mL medium in 35 mm Petri dishes with 20 mm glass-bottom central wells (Cellvis, Mountain View, CA, USA; Cat # D35–20-1.5-N) and subjected to treatment with dabrafenib plus trametinib (25 nmol/L /10 nmol/L) or vehicle treatment for 48 h. At 48 h of treatment, the culture media were removed, and the dishes were rinsed twice with 1 mL Dulbecco’s phosphate buffered saline (DPBS^+^ with calcium and magnesium) and then filled with 1 mL live cell imaging solution (LCIS^+^) (Molecular Probes, Eugene, OR, USA; supplemented with 11.1 mmol/L glucose and 2.0 mmol/L glutamine) one hour before ORI at the room temperature. Intracellular NADH and oxidized flavoproteins (Fp containing FAD) were measured using the ORI as described (14,22,23) with a Keyence inverted fluorescence microscope (BZ-X810, Keyence Corporation of America, Itasca, IL, U.S.A.). The cellular autofluorescence signals were imaged with a 40 W Light Emitting Diode (LED) source, a Plan Apochromat 20× objective (NA 0.75), a 2.83 million pixel monochrome 14-/8-bit CCD with an image resolution of 0.75 μm, matrix size 960×720, gain 6 dB, and bandpass excitation and emission filters (NADH: Ex 360/40 nm, Em 460/50 nm, exposure time 2.5 s; FAD: Ex 470/40 nm, Em 525/50 nm, exposure time 4 s). At least 3 fields of view (FOVs) were randomly selected and imaged for each dish. Raw fluorescence signals of NADH or FAD were subtracted with the background signals and thresholded at the SNR of 7.5 (SNR = subtracted signal/background standard deviation (SD)). Only pixels with signals above the SNR threshold were kept for further data processing. Optical redox ratios (ORR = Fp/(NADH+Fp)) were calculated on a pixel-by-pixel basis. Average NADH, Fp, and ORR were obtained by averaging across FOVs for each dish and then averaging across dishes within each treatment group. The experiment was performed in quadruple dishes.

### In Vivo Experiments

#### Tumor Production in Mice

Mice 4–6 weeks of age were purchased from Charles River Laboratories (https://www.criver.com/). YUMM1.7 cells were grown as monolayers at 37 °C in 5% CO2 in α-MEM medium. Tumor models were prepared by subcutaneous injection of 0.1 mL of a 500 K YUMM1.7 cells/mL suspension in Hank’s balanced salt solution into the right thigh of a male C57BL/6 black mice. YUMM1.7 melanoma tumor models were allowed to grow until the tumor volume reached ~250 mm^3^ and the tumor assumed a hemispherical shape before further study. Tumor dimensions were measured in three orthogonal directions and the tumor volume was calculated using the equation, V= π(l×w×d)/6, where l = length; w = width; d = depth of the tumor.

#### MR Spectroscopy Experiments

^31^P and ^1^H MRS experiments were performed on 10 male YUMM1.7 murine melanoma tumors with or without treatment [control: n=5, and dabrafenib plus trametinib treatment: *n* = 5)]. To prepare tumor-bearing mice for MRS experiments, 1% isoflurane in 1 L/min oxygen was used to anesthetize the mice. Animal respiration and temperature were monitored with a respiration pillow and rectal thermistor, respectively. The core temperature of the mice was maintained at 37 ± 1 °C by blowing warmed air into the bore of the magnet with heating controlled with a thermal regulator system (Small Animal Instruments Inc., Stony Brook, NY, USA). To measure the extracellular pH (pHe), 3-APP (300 mg/mL solution in water) was injected exogenously into the peritoneum of the mouse before placement in the spectrometer.

MRS experiments were performed on a Bruker 9.4 T 31 cm horizontal bore spectrometer using a homemade receiver coil. ^1^H and ^31^P MRS experiments were performed on YUMM1.7 melanoma tumors with and without treatment with dabrafenib and /or trametinib. MR spectroscopy data were acquired on Day 0, Day 2, and Day 5 with and without treatment. To detect lactate and alanine resonances and resolve overlapping lipid signals, ^1^H MRS was implemented with a slice-selective, double-frequency, Hadamard-selective, multiple quantum coherence (HDMD-Sel-MQC) transfer pulse sequence (24). Lactate and alanine concentrations were measured on Day 0, Day 2, and Day 5 using ^1^H MRS and positioning the tumor in a homemade single frequency (^1^H) slotted-tube resonator (15 mm in outer diameter, 13 mm in inner diameter, and 16.5 mm in depth). The acquisition parameters were set to TR = 4 s, 1024 data points, and 32 scans. In addition, a localized water spectrum was obtained using the same sequence without water suppression (TR = 4 s, 4 scans) serving as reference. Normalization to water signal is a standard practice in MRS quantification, as our group has previously demonstrated that normalization of metabolites to water effectively determines the absolute concentration of metabolites within a proportionality factor that depends on the degree of hydration of the tissue (25).

^31^P-MRS measurements were also conducted on these tumored mice. In brief, the tumor was placed in a custommade, dual-frequency (^1^H/^31^P) slotted-tube resonator with a 10 mm diameter. The Image-Selected in vivo Spectroscopy (ISIS) pulse sequence was used, with the following parameters: acquisition time (AT) of 64 ms, flip angle = 90°, TR = 2 s, spectral width (SW) = 7979 Hz, offset frequency = 430 Hz, and number of points 512 and 32 scans. The ^31^P MRS spectra were acquired with a decoupling duration of 64.16 ms, a decoupling power of 3.12 W, and a continuous phase decoupling (CPD) sequence element of 0.5 ms. A scout image was initially captured to align the acquisition voxel on tumors and reduce interference from external signals such as lipids and muscle.

All spectroscopic data were processed using NUTS (Acorn NMR Inc., Livermore, CA, USA) and MestReC software. In the acquired ^1^H spectra, the methyl signal peaks of lactate (1.33 ppm) and alanine (1.48 ppm) were integrated to obtain the area representing the signal intensity which were also normalized by the reference unsuppressed water signal intensity. Similarly, the ^31^P signals (PCr set at 0 ppm) from the β phosphate of NTP (βNTP) and inorganic phosphate (Pi) were integrated to calculate the βNTP/Pi ratio, and pHe and intracellular pH (pHi) values were calculated as described in our previous study (11,12,26). Additionally, for the total NAD(P)(H) measurement, a 40 Hz exponential filter was applied to enhance the apparent SNR of ^31^P MRS, and then the spectra from −5.5 to −11.5 ppm were fitted by modeling the peaks of αNTP (~−7.56 ppm), NAD(P)(H)+UDPG (approximately from −8 to −8.5 ppm), UDPG (~−9.8 ppm). Fitting of the upfield shoulder of αNTP extracted the combined signals of NAD(P)(H)+UDPG, and the subtraction of UDPG at ~−9.8 ppm from NAD(P)(H)+UDPG yielded the total NAD(P)(H) (=NAD^+^+NADH+NADPH, NADP^+^ concentration negligible) (27–30) (personal communication with Kevin E. Conley).

### Statistical Analysis

The data are presented graphically and summarized using bar graphs denoting the mean ± standard error (SEM) or standard deviation (SD). Independent paired *t*-test was performed for statistical analysis using SPSS 20. A *p*-value ≤ 0.05 was considered significant. Two-tail unpaired *t*-test with unequal variance was used in the analysis of the ORI data using Excel.

## RESULTS

**Figure 1.**
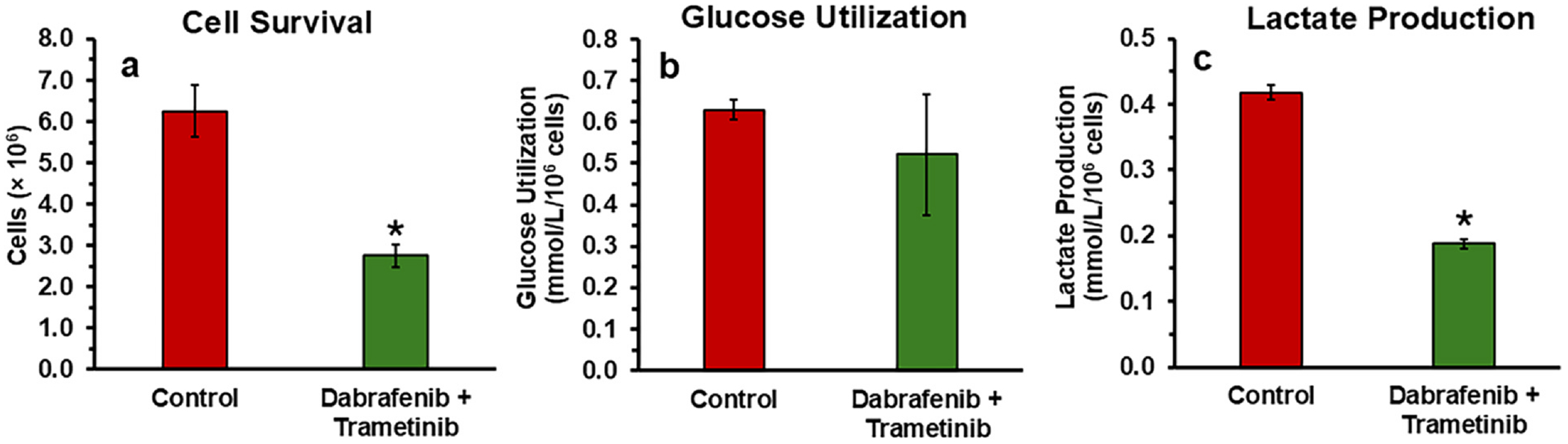
In vitro analysis of cell survival, glucose utilization and lactate production: **(a)** cell survival, **(b)** extracellular glucose consumption (measured via YSI biochemical analyzer), and **(c)** lactate production (measured via YSI biochemical analyzer) in cultured YUMM1.7 melanoma cell lines treated with dabrafenib (25 nmol/L) and trametinib (7 nmol/L) for 48 h, compared to untreated controls. Data in the figures are net changes of the mean ± SD (*n* = 3). Asterisks indicate a statistically significant change (*p* < 0.01) comparing metabolite concentrations of control vs. dabrafenib + trametinib.

**Figure 2.**
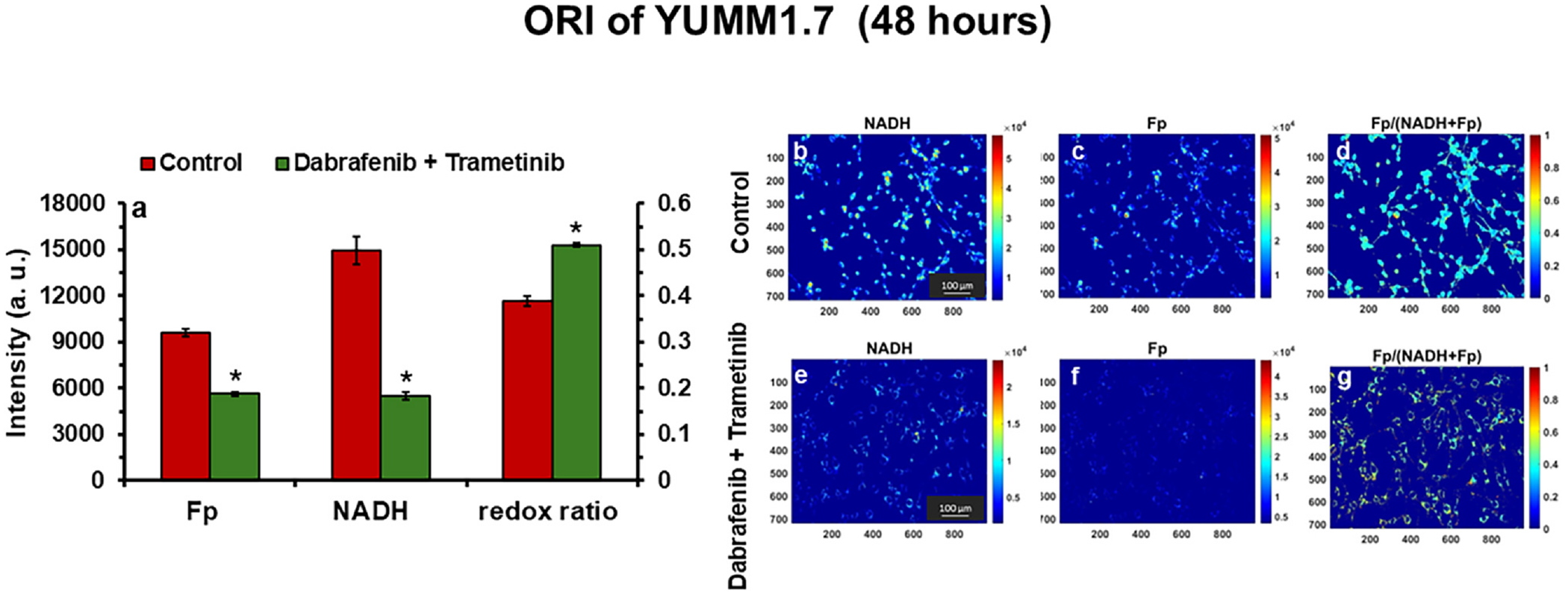
In vitro ORI analysis of YUMM1.7 cell cultures under treatment: **(a)** Optical redox imaging results indicate that combined dabrafenib and trametinib treatment significantly reduced NADH and Fp levels, while the optical redox ratio increased, highlighting a shift in cellular metabolism with treatment (mean ± SD, *n* = 4). Asterisks indicate a statistically significant change (*p* < 0.001) comparing metabolite concentrations of control vs. dabrafenib + trametinib. **(b–g)** Representative ORI images of NADH, Fp, and the redox ratio comparing the control and treatment groups.

The combination therapy of dabrafenib and trametinib significantly inhibited proliferation (~56%) of YUMM1.7 mouse melanoma cells within 48 h of treatment in vitro (Fig 1a). While glucose utilization remained relatively unchanged, lactate production decreased substantially by more than 50% (0.19 mmol/L vs. 0.42 mmol/L in controls) (Fig 1b and c). Notably, Fp and NADH levels were also decreased by 41% and 63%, respectively, leading to a 32% increase in the optical redox ratio (Fig 2). These findings suggest that the combined therapy induces metabolic reprogramming, characterized by reduced glycolytic flux and altered oxidative phosphorylation, ultimately leading to cell growth inhibition.

**Figure 3.**
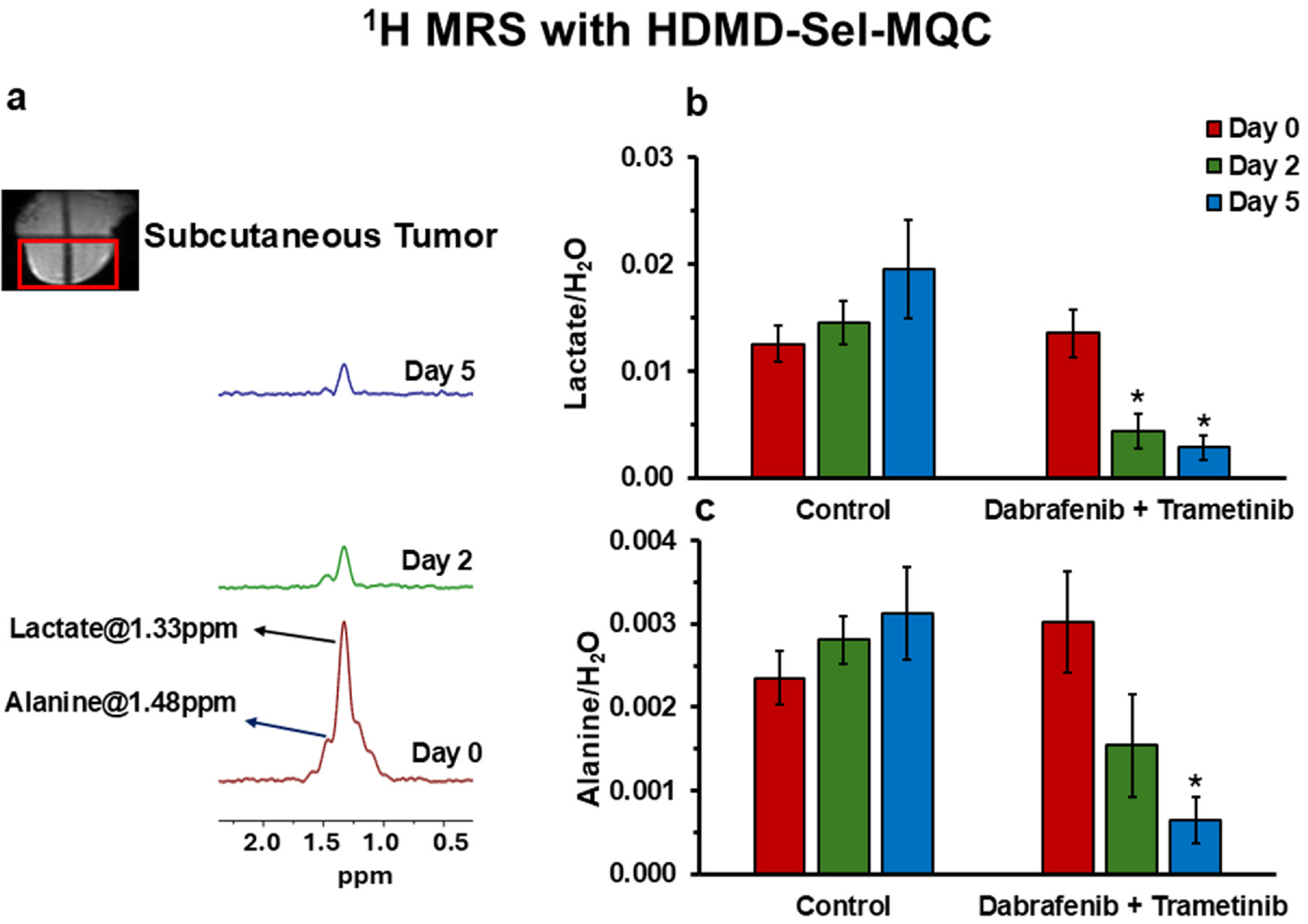
In vivo ^1^H MRS analysis of lactate and alanine: **(a)** Panel spectra using HDMD-Sel-MQC pulse sequence from a localized tumor volume (as shown in red box) in YUMM1.7 melanoma model in mice showing changes in the levels of tumor lactate and alanine on Day 0, Day 2, and Day 5 with dabrafenib (30 mg/kg) + trametinib (10 mg/kg) administration in the YUMM1.7 melanoma model. **(b–c)** Bar plots show lactate and alanine measurements (*n* = 5) at Day 0, 2, and Day 5 control and dabrafenib + trametinib treated groups. All data are shown as mean ± SEM, *significant differences compared to baseline (*p*-values < 0.05).

The in vivo ^1^H NMR spectroscopy measurements revealed distinct methyl resonances corresponding to lactate and alanine (Fig 3). Lactate, a glycolytic byproduct, serves as a reliable marker of glycolytic capacity in YUMM1.7 melanoma models. Treatment with dabrafenib and trametinib significantly reduced lactate levels between days 0 and 2, and between days 0 and 5 by 67% and 79%, respectively. This decrease indicates effective inhibition of lactate generation associated with the Warburg effect, a crucial metabolic phenotype for cancer cell proliferation and survival. While alanine levels also decreased, the most significant reduction occurred between days 0 and 5. This temporal pattern suggests a more gradual impact of the treatment on alanine metabolism compared to glycolysis. Alanine, a non-essential amino acid, can be synthesized from pyruvate, a glycolytic intermediate. Reduced levels of alanine might be associated with decreased availability of pyruvate secondary to inhibited glycolysis. Additionally, alanine could serve as a carbon source for energy production, especially under the condition of nutrient deprivation (31). A decrease in alanine levels may therefore reflect a metabolic adaptation in response to treatment. Overall, the inhibition of lactate generation and the decrease in alanine level over time may signify a metabolic shift towards a more oxidative phenotype.

**Figure 4.**
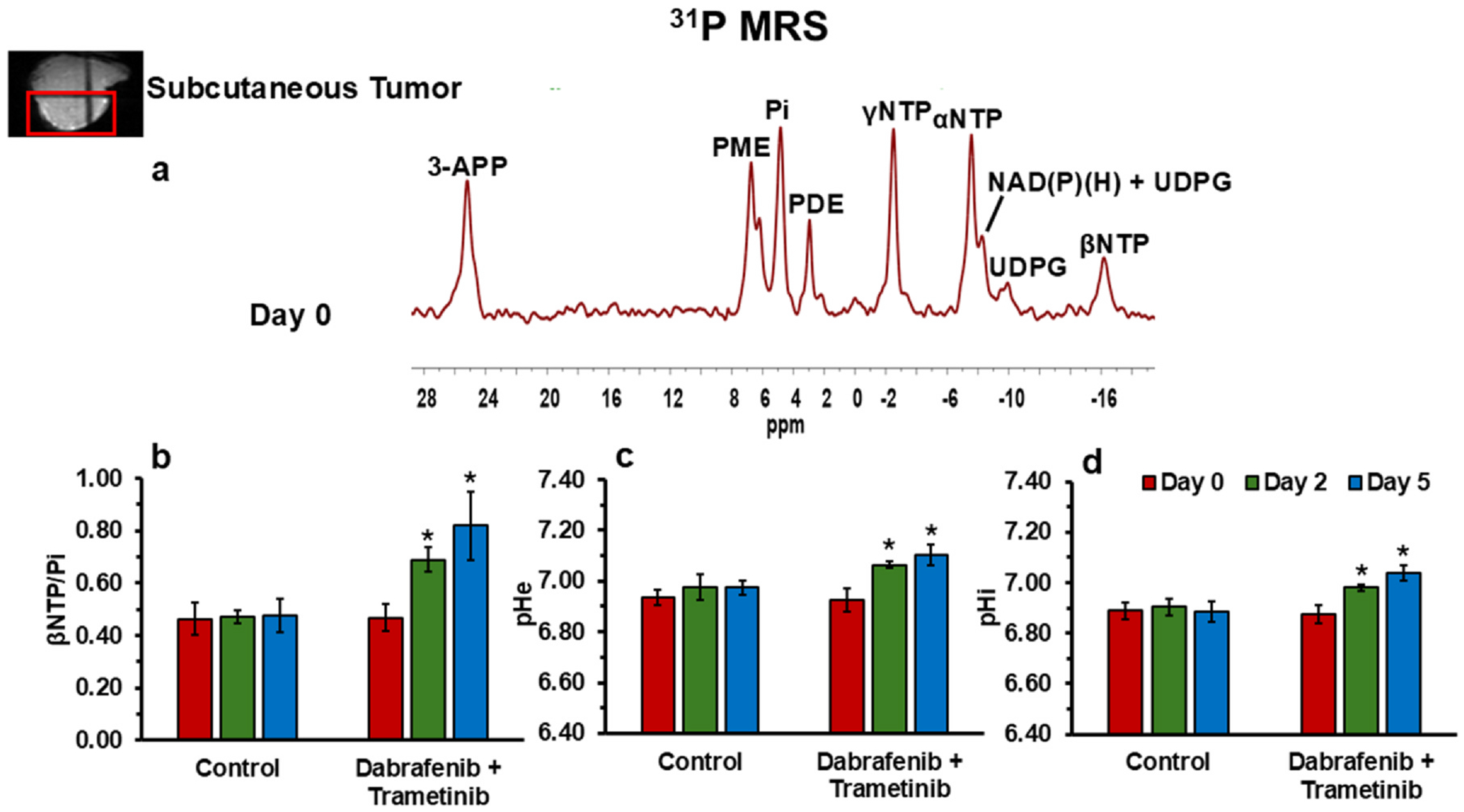
In vivo ^31^P MRS analysis of bioenergetics and pH: **(a)** Representative spectra of YUMM1.7 melanoma tumors from a localized volume (as shown in red box). **(b-d)** Comparisons of the bioenergetic index βNTP/Pi **(b)**, extracellular pH (pHe) **(c)**, and intracellular pH (pHi) **(d)** between Day 0, Day 2, and Day 5 in both control (*n* = 5) and treated (*n* = 5) groups. Data are presented as mean ± SEM, with * indicating statistically significant differences (*p* < 0.05) compared to baseline (day 0).

The ^31^P spectrum of the tumor models consists of well-resolved low-field resonances of βNTP and Pi (Fig 4a). The βNTP/Pi ratio, commonly used to represent the bioenergetic status, could be quantitated for YUMM1.7 model melanomas in control and treated groups (Fig 4b). We observed significant increase in the βNTP/Pi ratio of treated groups of YUMM1.7 melanoma tumors after treatment with dabrafenib plus trametinib at both two- and five-days following treatment. However, significant changes in pHe (Fig 4c) and pHi (Fig 4d) that show neutral or alkaline shifts were detected following treatment of YUMM1.7 melanoma with the combined treatment. These important findings are consistent with less lactate production and reduced acidification.

**Figure 5.**
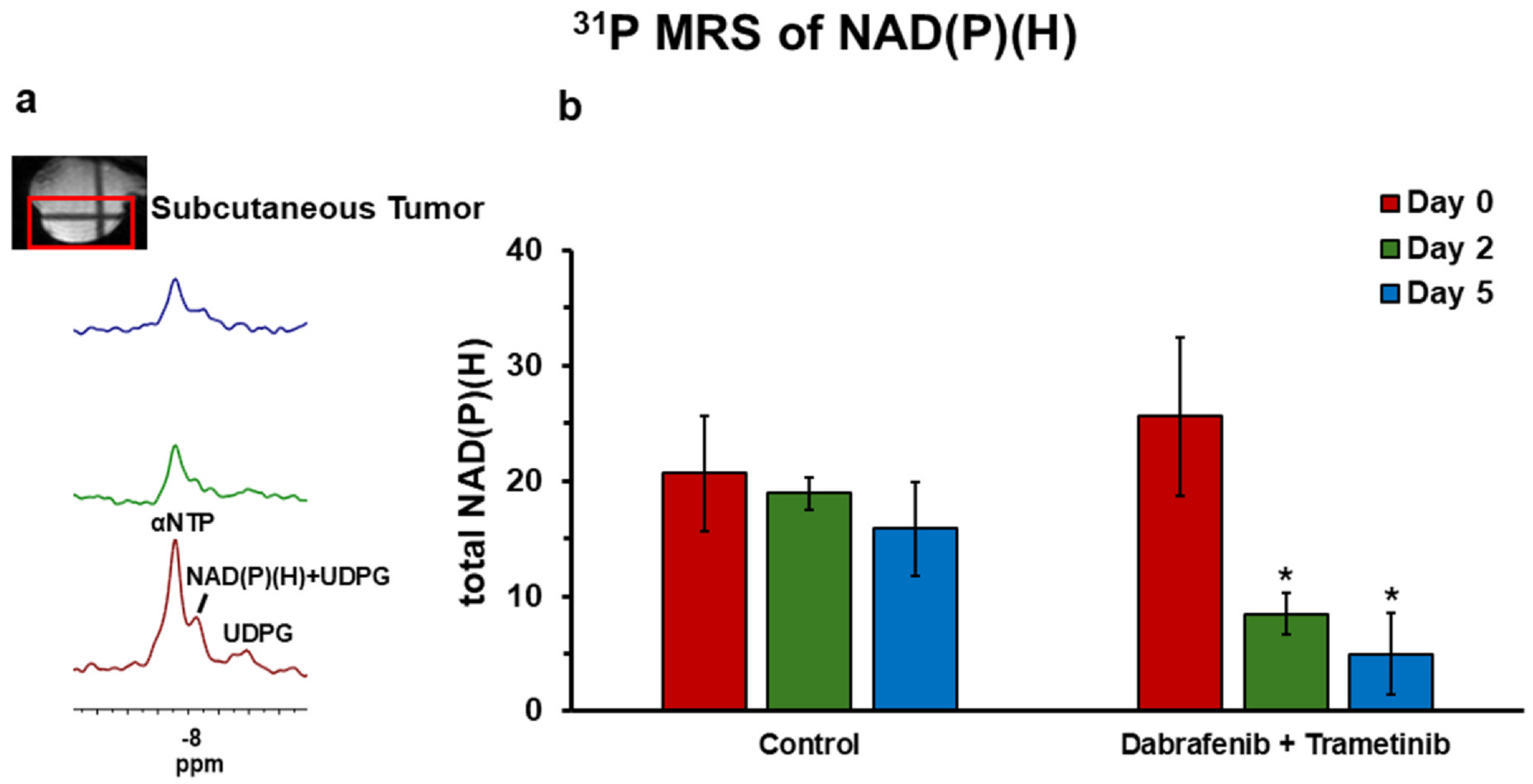
In vivo ^31^P MRS analysis of NAD(P)(H): **(a)** Representative spectra of YUMM1.7 melanoma tumors from a localized volume (as shown in red box) treated with combined dabrafenib (30 mg/kg) and trametinib (10 mg/kg) therapy. **(b)** Bar plot comparing total NAD(P)(H) levels at day 0, 2, and 5 in control (*n* = 5) and treated (*n* = 5) groups. Data presented as mean ± SEM, with * indicating statistically significant differences (*p* < 0.05) compared to Day 0.

Total NAD(P)(H) levels, quantified by ^31^P NMR spectroscopy, provide valuable insights into the cellular redox state and metabolic activity. The upfield shoulder of the ^31^P-MRS αNTP peak can be contributed by NAD(H) and NADP(H) as well (27,28) (Kevin E. Conley, personal communication). NADP(H), in equilibrium with NAD(H) via NADH transhydrogenase (32), is an important regulator of oxidative stress. A significant decrease in total NAD(P)(H) was observed in the treated YUMM1.7 melanoma tumors between days 0 and 2, and between days 0 and 5, while control groups exhibited non-significant changes (Fig 5). This reduction in total NAD(P)(H) indicates a decline in the cell’s redox capacity and metabolic activity.

**Figure 6.**
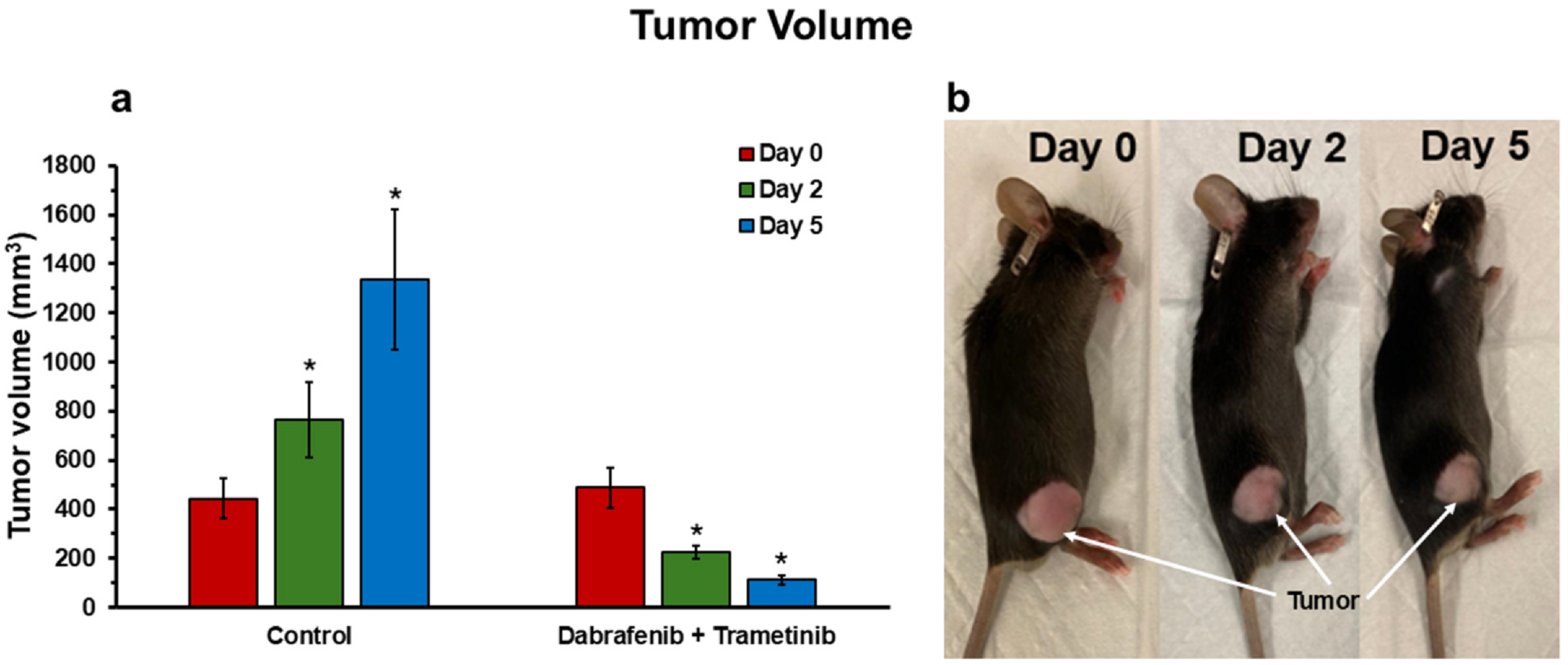
Tumor volume analysis under treatment. **(a)** Bar plot showing a significant increase in tumor volume in the control group (*n* = 5), whereas a significant decrease is observed in tumor volume for the treated group (dabrafenib + trametinib; *n* = 5) in YUMM1.7 melanoma models. **(b)** C57BL/6 black mice melanoma tumors (YUMM1.7) before (Day 0) and after treatment (Days 2 and 5) with combined dabrafenib and trametinib therapy. *Significant differences compared to the Day 0 baseline (*p*-values < 0.05).

As expected, control mice untreated with either drug, exhibited a significant monotonic increase in tumor volume with time (Fig 6). On the other hand, YUMM1.7 melanoma tumors exhibited a large decrease in tumor volume at days 2 and 5 following treatment with combined dabrafenib and trametinib relative to baseline (prior to treatment).

## DISCUSSION

### Probing Metabolic Reprogramming

The combination therapy of dabrafenib and trametinib exerts significant effects on the metabolic and proliferative characteristics of YUMM1.7 mouse melanoma cells. In the present study, the dual inhibition of BRAF and MEK signaling pathways resulted in a 56% inhibition of cellular proliferation within 48 h, demonstrating the profound cytostatic effect of this therapeutic strategy (Fig 1a). By targeting the MAPK pathway, the treatment not only disrupts key signaling cascades but also induces extensive metabolic reprogramming, likely contributing to its anti-tumor efficacy.

Our study demonstrates that combined treatment with dabrafenib and trametinib profoundly affects metabolic pathways in murine YUMM1.7 melanoma cells. Notable alterations were observed in key metabolites, including lactate, alanine, mitochondrial NADH, and total NAD(P)(H), accompanied by shifts in pHi and pHe as well as changes in cellular bioenergetics.

Despite stable glucose utilization, lactate production a key feature of the Warburg effect was significantly reduced by over 50% in treated YUMM1.7 melanoma cells (Fig 1c). This finding was corroborated by in vivo ^1^H MRS, which revealed lactate reductions of 43% and 79% at two- and five-days post-treatment, respectively (Fig 3b). These results indicate a rapid and pronounced inhibition of glycolysis and the Warburg effect, effectively disrupting the bioenergetic and biosynthetic processes and homeostasis essential for tumor survival and proliferation. Furthermore, the reduced lactate generation was associated with or resulted in neutral to alkaline pH shifts in both intracellular and extracellular compartments that support the disruption of tumor acidification (Fig 4c and d), impairing the TME conducive to invasion and therapeutic resistance. Alanine levels also decreased progressively, suggesting that the depletion of pyruvate, the substrate for alanine synthesis, limits the cells’ metabolic adaptability (Fig 3c).

Together, these findings suggest the combined therapy with dual inhibition of BRAF and MEK exerts significant effects on cancer cell metabolism through several possible mechanisms. It has been shown that BRAF mutations can activate MAPK and HIF-1α, leading to enhanced glycolysis and suppressed OXPHOS (33–36). The observed suppression of glycolytic activity, evidenced by reduced lactate production, is likely due to the inhibition of glycolytic enzymes regulated by the MAPK/ERK pathway. For example, BRAF/MEK dual inhibition is expected to deactivate ERK, which can regulate the expression and activity of phosphofructokinase in macrophage, which is a key regulator of glycolytic flux (37). However, the glucose utilization was not significantly different between control and treated cells in vitro in this study, which suggest the BRAF/MEK dual inhibition might act on downstream steps of glycolysis in melanoma cells. A possible mechanism involves a metabolic shift from glycolysis to mitochondrial OXPHOS, consistent with enhanced bioenergetic efficiency as evidenced by an increase in the βNTP/Pi ratio (Fig 4b) and a decrease in mitochondrial NADH (Fig 2). The optical redox ratio, derived from the balance of Fp (containing FAD) and NADH levels, also serves as a sensitive indicator of mitochondrial metabolic state. The 32% increase in this ratio after treatment suggests a metabolic state favoring mitochondrial activity and reduced anabolic processes, which are essential for cell proliferation. However, on the other hand, cancer therapy could impair mitochondrial enzyme expression and activity, leading to decreased respiration and reduced ATP synthesis (38,39). A partial shutdown of glycolysis and TCA cycle could happen that limits the raw materials needed for cell proliferation in addition to causing a respiration deficit. It remains to be investigated in the future whether the BRAF/MEK dual inhibition suppresses or enhances OXPHOS activity by more direct measurement of oxygen consumption rate.

The significant decline in mitochondrial NADH in vitro and total NAD(P)(H) levels in vivo (Figs. 2 and 5) could also highlight an impaired redox capacity in treated tumors. Although currently the ORI cannot differentiate NADPH from NADH (with identical fluorescence signature), the bound NADH in mitochondria is expected to dominate the in vitro ORI signal due to lower concentration and less quantum efficiency of NADPH (40,41). Due to insufficient SNR and close chemical shifts, ^31^P-MRS in this study cannot differentiate between NADPH, NAD^+^, and NADH (cellular NADP^+^ level is expected to be much less than NADPH and negligible). However, the information on total NAD(P)(H) is still valuable for estimating the redox capacity of tumors. NAD(H) is integral to maintaining glycolytic and mitochondrial metabolic fluxes and the redox homeostasis since they are in a dynamic equilibrium with NADPH, an important cellular reductant, via NADH transhydrogenase. Thus, the depletion of total NAD(P) (H) may indicate compromised energy metabolism, reduced redox capacity, and heightened oxidative stress.

These findings are consistent with previous studies (11,12) demonstrating the metabolic reprogramming and vulnerability of melanoma cells under MAPK pathway inhibition.

The observed changes in MRS-detectable biomarkers in YUMM1.7 tumors following combination treatment with dabrafenib and trametinib suggest a potential reduction in hypoxic conditions. This interpretation is supported by several key observations. The increase in both pHi and pHe indicates a shift away from the acidic environment typically associated with hypoxic tumors, which results from increased glycolysis and lactate production (42–45). Similarly, the reduction in total lactate levels supports a shift away from hypoxia-induced glycolysis. Furthermore, the decrease in alanine levels, a byproduct of glucose metabolism under hypoxic conditions, suggests reduced anaerobic metabolism associated with hypoxia (42–45). The observed reduction in total NAD(P)H, which is critical for maintaining redox balance and supporting biosynthetic processes upregulated in cancer cells, further reinforces the interpretation of decreased hypoxia-induced metabolic adaptations (42–45). These metabolic changes align with the known effects of dabrafenib and trametinib, which target the MAPK pathway and influence tumor metabolism and microenvironment, potentially contributing to a reduction in hypoxic conditions (44,45). Furthermore, we will consider hypoxia-specific experiments, such as immunohistochemical staining for hypoxia markers (e.g., HIF-1α) (46,47) to assess the presence of hypoxic regions in YUMM1.7 tumors (48,49) and their role in metabolic reprogramming for future study.

### Potential Early Biomarkers and Translational Feasibility

Metabolic changes often precede measurable reductions in tumor volume, highlighting their potential utility as early biomarkers for monitoring therapeutic efficacy. The rapid and significant declines in lactate and alanine levels following dabrafenib and trametinib treatment suggest their utility in monitoring therapeutic responses. Combined with bioenergetic indices like βNTP/Pi ratios and pH alterations, these metabolic shifts could serve as noninvasive, real-time biomarkers, enabling better stratification of responders and adjustment of therapeutic regimens.

The translational feasibility of these biomarkers is promising, given the increasing accessibility of advanced imaging modalities like ^1^H/^31^P MRS and their potential for integration into clinical workflows (50–52). Utilizing these markers in human melanoma could facilitate the development of personalized treatment strategies, particularly in cases where conventional imaging is challenging to accurately assess tumor burden.

### Limitations and Future Directions

While this study highlights the profound metabolic reprogramming induced by dabrafenib and trametinib, several limitations warrant attention. First, this study with a focus on utilities of imaging techniques and potential biomarker development, does not provide clear molecular mechanisms underlying the functioning of combination therapy. Future research could focus on targeted metabolomics and genetic approaches to further elucidate the effects of combined dabrafenib and trametinib therapy. Examining alterations in metabolic enzymes and transporters could reveal how these drugs reprogram cellular metabolism, while genetic modulation of key pathways may identify critical mediators of trametinib’s effects that remain unclear so far. Identifying molecular mechanisms driving resistance and leveraging them to develop combinatorial strategies will be crucial. Advanced metabolomics and imaging techniques, including MRS, with its sensitivity to detect metabolic changes, and Positron Emission Tomography (PET), which enables real-time assessment of metabolic fluxes, can aid in elucidating these mechanisms and refining biomarker panels for clinical applications. Second, the findings are derived from a specific preclinical model, which may not fully represent the metabolic heterogeneity of human melanomas. Extending these results to patient-derived models and clinical settings is necessary to validate their translational applicability. Third, the focus on short-term metabolic effects limits insights into long-term outcomes, including tumor progression, recurrence, and resistance. Future research will incorporate longitudinal analyses to explore these dynamics comprehensively. Fourth, the potential off-target effects of the therapy remain unexplored and could influence metabolic and therapeutic outcomes. Exploring combination therapies that exploit identified metabolic vulnerabilities, such as targeting OXPHOS, could further enhance therapeutic efficacy. Lastly, additional investigations into the TME, including pH, pO2, and immune and stromal interactions, could provide a holistic understanding of treatment impacts.

## CONCLUSIONS

In conclusion, consistent with previous studies (11,12), dual BRAF/MEK inhibition with dabrafenib and trametinib induces profound metabolic reprogramming in YUMM1.7 melanoma cells. The marked reductions in the levels of lactate, alanine, and mitochondrial NADH, coupled with stable bioenergetics and pH shifts, signify a suppression of glycolytic activity and a metabolic transition toward oxidative phosphorylation. The reduced NADH and total NAD(P)(H) also indicate suppressed redox capacity in tumor tissue. These findings reveal a dual mechanism of action: disruption of oncogenic signaling pathways and targeted modulation of tumor metabolism. These metabolic reprogramming compromises tumor growth and survival, as evidenced by the significant reduction in tumor volume. It remains to be studied to understand how this metabolic reprogramming specifically regulates the tumor progression and response and resistance to treatment in the future. Furthermore, this study underscores the potential of metabolic biomarkers lactate, alanine, pH, and bioenergetic indices including NTP and NAD(P)(H) as powerful, noninvasive tools for monitoring therapeutic efficacy. These discoveries provide a deeper understanding of metabolic reprogramming and possible vulnerabilities in melanoma that may open new avenues for optimizing targeted therapies to improve clinical outcomes.

## Data Availability

The original datasets analyzed during the current study are available from the corresponding author upon reasonable request.

## Abbreviations:

MRS: Magnetic Resonance Spectroscopy
ORI: Optical Redox Imaging
ORR: Optical Redox Ratio
OXPHOS: oxidative phosphorylation
TME: Tumor Microenvironment
